# Time‐dependent neuronal changes associated with craving in opioid dependence: an fMRI study

**DOI:** 10.1111/adb.12554

**Published:** 2017-09-22

**Authors:** Anna Murphy, Dan I. Lubman, Shane McKie, Prun S. Bijral, Lesley A. Peters, Qasim Faiz, Sophie E. Holmes, Ian M. Anderson, Bill Deakin, Rebecca Elliott

**Affiliations:** ^1^ Neuroscience and Psychiatry Unit, Faculty of Biology, Medicine and Health University of Manchester UK; ^2^ Department of Biological Sciences University of Huddersfield UK; ^3^ Turning Point Eastern Health Australia; ^4^ Eastern Health Clinical School Monash University Australia; ^5^ Chapman‐Barker Unit Greater Manchester West NHS Foundation Trust UK; ^6^ Change, Grow, Live UK; ^7^ Pennine Care NHS Foundation Trust UK; ^8^ DISC, My Recovery Tameside UK; ^9^ Department of Psychiatry Yale University School of Medicine West Haven CT USA; ^10^ Greater Manchester Mental Health NHS Foundation Trust UK

**Keywords:** addiction, craving, cues, fMRI, heroin, opioid

## Abstract

Relapse after initially successful treatment is a significant problem facing the treatment of opioid dependence. Evidence suggests craving elicited by re‐exposure to drug cues may precipitate relapse. Attempts to identify neural biomarkers of cue‐elicited craving have yielded inconsistent findings. We aimed to apply a novel continuous functional magnetic resonance imaging technique to follow the minute‐to‐minute evolution of brain responses, which correlate with the waxing and waning of craving. Newly detoxified male opioid‐dependent patients and healthy control participants attended two separate, counterbalanced, functional magnetic resonance imaging scanning sessions during which they viewed a 10‐minute video (drug cue or neutral cue) followed by 5 minutes of fixation. Participants rated the intensity of their craving throughout each session. We hypothesized that subcortical/ventral prefrontal cortex (PFC) regions and dorsal PFC regions would show different associations with craving reflecting their putative roles in appetitive processing versus cognitive control. Compared with controls, drug cue (minus neutral cue) video recruited the left amygdala and was temporally correlated with craving. In contrast, dorsal anterior cingulate blood‐oxygen‐level‐dependent signal time course was higher than controls only during a period after cue exposure when craving levels were declining. Against expectations, neither the ventral striatum nor ventral PFC was significantly recruited by drug cue exposure. Findings suggest that the amygdala has a central role in craving, whereas the dorsal anterior cingulate may control craving in treatment‐seeking patients. Time course analysis yielded new insights into the neural substrates of craving that could objectively validate development of psychological and pharmacological approaches to sustained abstinence.

## Introduction

Neural and psychological responses to stimuli or contexts previously associated with drug reward (i.e. drug cues) play a significant role in drug dependence (Lubman, Yucel, & Pantelis 2004; Epstein *et al*. 2009). Conditioning mechanisms increase the incentive value of these cues, creating powerful secondary reinforcers that can capture attention and elicit craving (Tiffany & Wray 2012), and contribute to further drug seeking and drug taking. Consistent with this notion, several studies have reported that craving measured before or during treatment can predict subsequent relapse (Weiss *et al*. 2003; McHugh *et al*. 2014), whilst ‘real‐time’ craving and cue exposure have been shown to immediately precede relapse and drug use (Epstein *et al*. 2009; Moore *et al*. 2014; Fatseas *et al*. 2015). Functional magnetic resonance imaging (fMRI) studies have attempted to identify brain systems that mediate cue‐elicited craving (Zijlstra *et al*. 2009; Li *et al*. 2012; Jasinska *et al*. 2014). Two recent meta‐analyses of these studies (Chase *et al*. 2011; Kuhn & Gallinat 2011) report reliable activation to drug cues within regions including the amygdala and ventral striatum, which are important for processing reward‐related stimuli and motivation (Haber & Knutson 2010), and medial prefrontal regions. These include the ventral medial prefrontal cortex (VmPFC) (Chase *et al*. 2011), which is involved in the representation of reward value and reward‐related decision making (Elliott, Agnew, & Deakin 2008; Grabenhorst & Rolls 2011), and the anterior cingulate cortex (Kuhn & Gallinat 2011), which is important for reward and error‐related behavioural and cognitive control (Shenhav, Botvinick, & Cohen 2013). Although there are some consistencies in the regions that are activated by drug cues, there is little evidence that any reliably correlate with craving (Tiffany & Wray 2012). This suggests the typical design of cue‐elicited craving studies, that measure brief presentations of drug‐related images interspersed with neutral control images, identify immediate brain responses to drug cues, rather than craving per se. Video imagery of drug‐related activities provides contextually richer and more realistic cues that better model real‐world exposure and elicit strong craving responses (Childress *et al*. 1999; Tong, Bovbjerg, & Erblich 2007). Videos have been extensively used to follow psychophysiological responses to craving although this has been limited to box‐car on‐off fMRI designs through the lack of techniques or tradition for following blood‐oxygen‐level‐dependent (BOLD) responses over minutes. We borrowed pharmaco‐MRI methods developed to follow and localize the action of drugs on the CNS, to follow the effect of sustained craving elicited by video cues, in hypothesized regions of interest. We reasoned that this approach could capitalize on the video mode of drug cue delivery, providing time course fMRI information for investigation of how psychological variables relate to brain response over time. We applied this approach in recently abstinent individuals with opioid dependence (OD), to better delineate brain responses related to the experience of craving over a period of cue exposure. We additionally investigated brain response for a period after cue exposure to gain insights into regions that may be involved in cognitive/behavioural control. Although videos have previously been used in fMRI studies of substance dependence, to our knowledge, dynamic brain responses to these cues have not previously been explored.

In recovering dependent individuals, brain responses to drug cues may reflect not only craving‐related activity but also attempts to control craving (Goldstein & Volkow 2011). This may explain why some participants in previous cue exposure studies have not reported significant overall changes in craving scores (Zijlstra *et al*. 2009; Wang *et al*. 2011). Time course information derived dynamically across video stimuli could help distinguish briefly induced craving and subsequent control at a neuronal level, thereby disambiguating distinct processes that may have been compounded in previous studies. We hypothesized that early development of craving would be associated with activation of amygdala, ventral striatum and ventral medial prefrontal regions involved in processing reward value and incentive motivation. In our treatment‐seeking sample, with long‐term goals of abstinence, we further hypothesized that response in the dorsal anterior cingulate cortex would develop both during and after the video as subjective craving decreased, reflecting conscious control mechanisms.

## Methods and Materials

### Participants

#### Opioid‐dependent individuals (n = 18)

This study was approved by the NHS Research Ethics Committee. Participants with OD were recruited from a residential detoxification unit within Greater Manchester (see Table 1 for demographic information). Treatment involved a 4‐week residential stay with a 10‐day methadone stabilization and reduction, followed by 10 days of lofexidine treatment, an alpha‐2 adrenergic receptor agonist used to treat opioid withdrawal symptoms. Participants took part in the study during the last week of their residential stay when they were opioid free (confirmed by urine drug screen). Lofexidine was extended at low doses for participants still experiencing withdrawal symptoms during this final week, and some ODs were receiving other psychoactive medications as indicated by Supporting Information Table **S1**. The OD group was recruited according to the following inclusion criteria: male, aged 18–55; current DSM IV heroin dependence (the majority of patients also met criteria for current or past dependence on other substances; Table 1); no history of serious psychiatric, neurological or physical illness; no contraindications for MRI; and IQ of ≥85 [determined using the Wechsler Test of Adult Reading (Holdnack 2001)]. Participants were identified and approached by members of the clinical team on the basis of detailed physical and mental health assessments on admission to the unit. All participants meeting the inclusion criteria were approached to take part in the study. A total of 39 patients were enrolled in the craving study; however, 19 were discharged from the unit before any testing could take place (mainly self‐discharge or early discharge due to use of prohibited substances). Twenty patients were scanned as part of the study, although two were excluded from analysis because of excessive movement (see Preprocessing section for further details), leaving *n* = 18 who were included in analysis. Handedness was assessed with the Edinburgh Handedness Inventory (Oldfield 1971).

**Table 1:** Demographic and clinical information of OD (*n* = 18) and HC (*n* = 20) groups included in the analysis.

	OD group	HC group	*p*‐value
Age	34.8 (6.3)	31.6 (7.2)	0.15
IQ	103.1.9 (7.7)	114.1 (4.7)	<0.05
Years of education	11.5 (2.2)	16.5 (3.3)	<0.05
No. of right handers	16 (89 percent)	18 (90 percent)	0.91
No. of smokers (nicotine)	13 (72 percent)	9 (45 percent)	0.09
Clinical Variables for OD group			
Years of opioid dependence	12.8 (7.7)	—	
Average daily opioid use (g)	0.7 (0.6)	—	
Age of first use (years)	20.3 (4.3)		
No. who primarily smoke/inject/ or use both methods for heroin administration	5 (28 percent)/5 (28 percent)/8 (44 percent)	—	
No. of OD group with current/past history of:		—	
Alcohol dependence	7 (39 percent)	—	
Cocaine dependence	7 (39 percent)		
Amphetamine dependence	1 (6 percent)	—	
Benzodiazepine dependence	3 (17 percent)	—	
Heroin dependence only (except nicotine)	6 (33 percent)	—	

Age, IQ, years of education, years of dependence, average daily amount used and age of first use expressed as mean and standard deviation (in brackets). All other variables expressed as frequency and proportion of group (in brackets). *P*‐values taken from independent *t*‐tests for continuous data and chi‐squared tests for frequency data.

#### Healthy controls (n = 20)

Healthy controls (HCs) were recruited who were familiar with graphic scenes of drug use because of occupational experience (e.g. drug treatment workers and other health professionals). The HC group met the same inclusion criteria outlined previously and additionally had no current or past history of drug dependence or misuse (except nicotine) and had never used heroin in their lifetime. No healthy volunteers were receiving psychoactive medications. Participants were screened with the Mini International Neuropsychiatric Interview (Sheehan *et al*. 1998), Wechsler Test of Adult Reading and Edinburgh Handedness Inventory.

All participants provided written consent and were paid for their participation with vouchers.

### Procedures

**Figure 1:**
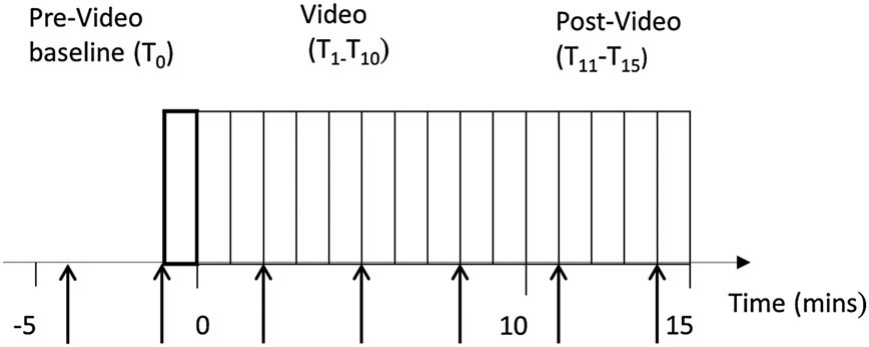
Diagram illustrating 1‐minute time‐bins for the drug cue and neutral sessions. The 480 scans were divided into 1 minute time‐bins. T_0_ was the 5th minute of the pre‐video baseline, T_1_–T_10_ were the time‐bins acquired during the video and T_11_‐T_15_ were the post‐video time‐bins. The arrows indicate the time points of the presentation of the Likert‐like subjective rating scales

Participants attended two separate, counterbalanced sessions. Patients were scanned in the last week of their residential stay when they were opioid free. On each occasion, participants underwent fMRI, once whilst viewing a 10‐minute heroin video (drug cue session) and once whilst viewing a 10‐minute video containing scenes matched for visual complexity but lacking emotionally arousing content (neutral session). Videos were preceded and followed by 5 minutes of fixation (Fig. 1). Images were presented using e‐prime (Psychology Software Tools, Inc, Sharpsburg, USA) (version 2.0).

### Measures

Participants were presented with Likert‐like subjective rating scales throughout the scanning session. Participants were prompted to respond by a screen displaying the following text ‘do you feel you are …’, which was presented for 3 seconds before slides stating ‘Craving?’, ‘Withdrawing?’, ‘Anxious?’ or ‘High?’, were presented for 4 seconds along with the following scale: 1 (not all), 2 (slightly), 3 (moderately) and 4 (extremely). Participants responded via a button box. The scales were presented at 3‐minute intervals in each session (Fig. 1)

### Behavioural data

Mixed ANOVAs were carried out for each of the four subjective rating scales with time of rating (six levels including a pre‐video baseline rating, three during the video and two post‐video ratings) and session (drug cue, neutral) as within‐subject factors and group (HC, OD) as the between‐subject factor. Session order was included as a covariate. The pre‐video rating taken during the last minute of the baseline period (T_0_) was used as the pre‐video baseline rating. A Greenhouse‐Geisser correction was applied when Mauchley's assumption of sphericity was violated. Ratings from one HC were not included as the participant could not see the rating scales.

### Scanning and analysis

Whole brain T2* weighted images were acquired on a 3 Tesla Philips *Achieva* scanner with single shot, multi‐slice echo planar (EPI) pulse sequence. Each volume comprised 34 contiguous axial slices (TR = 2.5 s, TE = 35 ms, 96 × 96 matrix, in‐plane voxel size 3.0 mm × 3.0 mm, slice thickness 3.5 mm). A high‐resolution T1‐weighted structural image was also acquired for each participant for coregistration during preprocessing and to exclude any structural abnormality.

#### Preprocessing

Functional data were analysed using statistical parametric mapping (SPM8; The Wellcome Department of Cognitive Neurology, London, UK) running in MATLAB (Matlab, R2012a; Mathworks, Natick, Massachusetts, U.S.A.). Functional images were realigned and coregistered with the T1 structural image before normalization of the functional images into standard space was carried out using the unified segmentation approach (Ashburner & Friston 2005). Normalized images were smoothed using an 8‐mm Gaussian kernel. The smoothed functional images of the participants who moved more than 1 voxel were repaired by an interpolation method using the artefact repair toolbox (http://cibsr.stanford.edu/tools/ArtRepair/ArtRepair.htm). Two ODs with excessive movement were excluded (>10 percent volumes needing repairing). Eighteen ODs and 20 HCs were entered into subsequent analyses.

#### First‐level analysis

For both the drug cue and the neutral session, data were acquired continuously for 20 minutes (5 minutes before the start of each video to allow participants to acclimatize to the scanner environment, 10 minutes during presentation of the video and an additional 5 minutes after the end of the video) (Fig. 1). The resultant 480 acquired scans were divided into 1‐minute time‐bins (20 time‐bins in total). The 5th minute of the pre‐video baseline was treated as the baseline time‐bin T_0_, with each subsequent time‐bin (T_1–15_) compared with T_0_ using regression analysis within a general linear model framework. The exact shape of the signal of interest was unknown; therefore, no high‐pass filter was used. The neutral session averages were subtracted from the time equivalent drug cue session averages, resulting in ‘drug cue session–neutral session’ contrast images.

#### Region‐of‐interest analysis

A region‐of‐interest (ROI) approach was used to extract time course signals from key brain regions involved in drug cue reactivity based on recent meta‐analyses (Chase *et al*. 2011; Kuhn & Gallinat 2011). These composed of spheres of 7‐mm radius centred around the bilateral ventral striatum, amygdala and VmPFC, coordinates from Chase *et al*. (2011), and anterior cingulate cortex, coordinates from Kuhn & Gallinat (2011), as displayed in Fig. 3. Mean drug cue‐neutral session contrast estimates were extracted from each of the six ROIs using the Easy ROI toolbox implemented in SPM (http://www.sbirc.ed.ac.uk/LCL/LCL_M1.html) and entered into mixed ANOVAs in SPSS (version 20) to investigate effects of time and group on drug cue‐neutral session BOLD signal. Investigations were carried out for the whole session (T_0_ to T_15_) and for the video time‐bins (T_1_–T_10_) and post‐video time‐bins separately (T_11_ to T_15_) with session order included as a covariate. A Greenhouse‐Geisser correction was applied when Mauchley's assumption of sphericity was violated. A Bonferroni correction for the six separate ROIs results in a significance value of *p* < 0.008 for this analysis. However, this is a conservative approach, which may result in false negatives. Results are therefore reported significant at *p* < 0.05 with results surviving Bonferroni correction highlighted.

#### ROI BOLD signal time course and subjective rating time course temporal correlation analysis

In participants reporting a subjective rating change in response to the drug cue session, we investigated whether their subjective rating time course was temporally correlated with fMRI signal in the ROIs. Analyses were restricted to subjective ratings and ROI BOLD signal time courses, demonstrating significant effects in the ANOVAs. Before correlations were carried out, the pre‐video baseline subjective rating was subtracted from each video and post‐video rating for each session. The neutral session ratings were then subtracted from the drug cue session ratings. This was to ensure both the fMRI and subjective rating data reflected drug cue session changes from baseline minus neutral session changes from baseline (fMRI data already in this format after first‐level analysis). Finally, the fMRI BOLD signal data were averaged over 3‐minute intervals to reflect the 3‐minute interval of subjective rating presentation during each session. Correlational analyses were carried out between each individual's subjective rating and ROI BOLD signal time courses using Pearson's product moment correlation coefficient. Resultant correlation coefficients were Fisher's *z*‐transformed and entered into one‐sample *t*‐tests to determine group level significance.

## Results

### Subjective ratings

#### Craving

**Figure 2:**
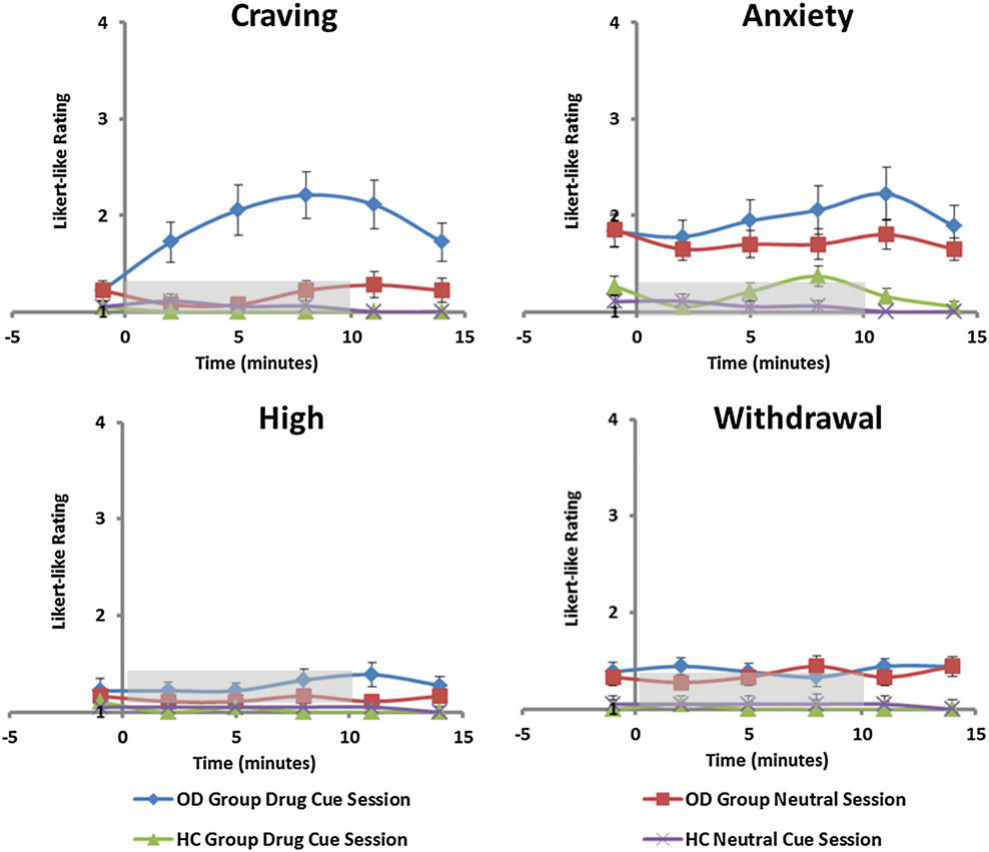
Subjective ratings taken during both the drug cue and neutral session in ODs (*n* = 18) and HCs (*n* = 20). Ratings were taken every 3 minutes throughout each session: 1 (not at all), 2 (slight), 3 (moderate) and 4 (extreme). The video started at 0 minute and ended at 10 minutes (grey bar). Error bars show standard error of the mean (SEM)

All main effects and two‐way interactions were significant (*p* < 0.001) as was the time × session × group interaction [F(3.54, 123.93) = 7.54, *p* < 0.001]. Subjective craving increased significantly during the drug session in the OD group only as shown in Fig. 2.

#### Anxiety

No significant main effect of time or session was revealed, although an overall main effect of group was found [F(1,34) = 23.45, *p* < 0.001] indicating higher anxiety in the OD than HC group (Fig. 2). A significant time × session interaction was found [F(3.53, 119.97) = 2.87, *p* = 0.03], but no three‐way interaction (*p* > 0.05); anxiety increased in both the OD and HC groups during the craving compared with neutral videos.

#### High

No significant main effects or interactions were found (Fig. 2).

#### Withdrawal

There was a significant main effect of group [F(1, 34) = 10.47, *p* = 0.003], but no other significant main effects or interactions. Higher withdrawal symptoms were reported in the OD group compared with HC group throughout both sessions (Fig. 2).

### Effects of group and time on ROI BOLD signal

**Figure 3:**
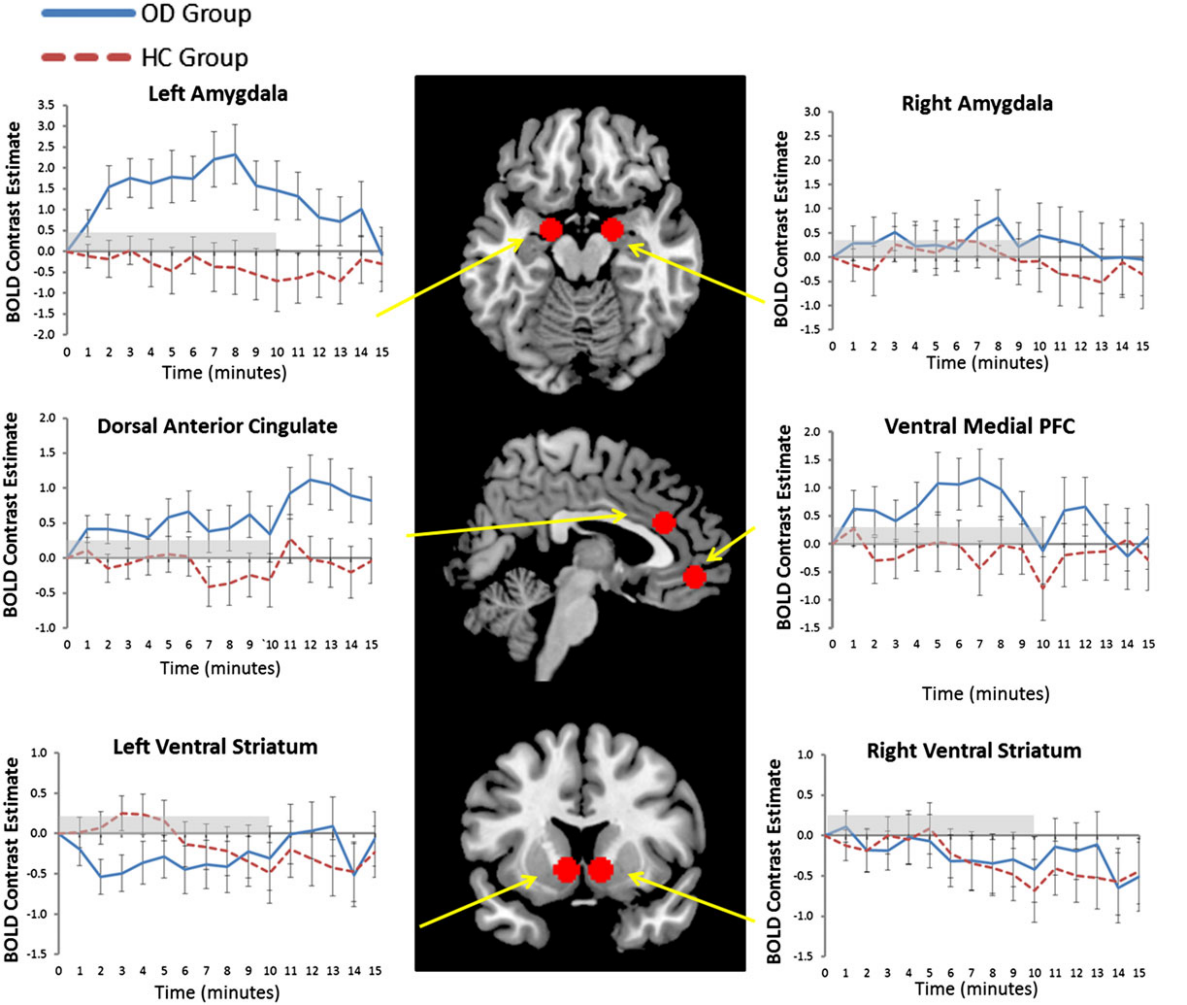
Regions of interest and average BOLD signal time course of each region. Time courses show the BOLD signal difference between drug cue‐neutral session in the bilateral amygdala, dorsal anterior cingulate and ventral medial prefrontal cortex. The videos were displayed from 0 to 10 minutes (grey bar). The last 5 minutes are the post‐video period. The solid blue bars demonstrate the average time course of the OD group (*n* = 18), and the red dotted line shows the average time course of the HC group (*n* = 20). Error bars show standard error of the mean (SEM)

Significant effects were observed only in the left amygdala and dACC (see Supporting Information Tables S2–S4 for *F*‐values and *p*‐values for all of the regions of interest). Within the left amygdala, there was an overall main effect of group (OD > HC, *p =* 0.014). Focusing on the video period only, there was a significant time × group interaction (*p* = 0.017) and a significant effect of group surviving Bonferroni correction for the six ROIs (*p* = 0.007). Figure 3 shows that these effects were due to amygdala BOLD signal increasing during the video in the OD group only. There was a significant effect of time (*p* = 0.041) and time × group interaction (*p* = 0.018) during the 5‐minute period after the end of the video. The effect of group was no longer significant during this period. Figure 3 illustrates that these effects were due to BOLD signal dropping in the OD group only after the end of the video session.

Within the dACC, there was a significant main effect of time for the entire drug cue‐neutral session time course, surviving Bonferroni correction for the six ROIs (*p* = 0.004). There was no time × group interaction, suggesting significant time course effects in both groups. dACC BOLD signal within the OD group was significantly higher than in the HC group within the post‐video period only (*p* = 0.046). Figure 3 illustrates that this effect is due to sustained recruitment of the dACC after the end of the video session in the OD group only.

There were no main effects or interactions for the four other ROIs examined.

### Temporal correlations between subjective ratings and ROI BOLD signal time courses

Analyses were restricted to craving/anxiety ratings and amygdala/dACC ROIs in the OD group, and anxiety ratings and dACC in the HC group, as these were the only ratings/ROI BOLD signal time courses that were significant in the ANOVAs. As some participants did not report a drug cue‐neutral session change in their subjective ratings (5 in OD group for craving, 4 in OD group for anxiety and 10 in HC group for anxiety), subjective rating‐BOLD signal correlations could not be conducted for these individuals for these rating scales.

The only correlation that was significant was between craving and left amygdala time courses in the OD group [mean (SD) Fisher's *z*‐score = 0.44(66), *t*(12)=2.40, *p* = 0.034) (Table 2).

**Table 2:** Mean and standard deviation of the *z*‐scores derived from Fisher's *z*‐transformation of correlation coefficients obtained from the ROI/subjective rating time course correlational analysis.

Correlations (scale‐ROI)	Mean (SD) *z*‐score	t(df)	*p*‐value
OD: Craving‐left amygdala	0.44 (0.66)	2.40(12)	0.034*
OD: Craving‐dACC	0.32 (0.71)	1.64(12)	0.126
OD: Anxiety‐left amygdala	0.15 (0.34)	1.56(13)	0.143
OD: Anxiety‐dACC	0.34 (0.99)	1.29(13)	0.220
HC: Anxiety‐dACC	0.006 (0.92)	0.02(8)	0.99

Table shows the *t*‐values obtained from one‐sample *t*‐tests and corresponding statistical significance.

* Significant at *p* < 0.05.

## Discussion

In this study, we demonstrated that a drug cue video increased craving in 13 of 18 recently detoxified opioid‐dependent participants, with a time course that correlated with BOLD signal time course within the left amygdala but not the dACC. Peak amygdala response occurred during the drug cue video in the OD group, whereas dACC BOLD responses peaked in the 5 minutes following the drug cue video (when craving levels were decreasing). Although anxiety ratings increased in response to the drug cue video, no correlations were found with either the amygdala or dACC. Against expectations, the ventral striatum was not recruited by the drug cue paradigm.

### The amygdala and craving

The significant correlation between amygdala BOLD signal and the time course of craving (but not anxiety) suggests the amygdala has a key role in the generation of cue‐elicited craving. This finding is in agreement with evidence demonstrating the amygdala to be important for Pavlovian‐conditioned behaviour and motivation (Janak & Tye 2015). Preclinical findings demonstrate both the basolateral and central amygdala to be crucial for generating drug‐seeking responses to drug cues (Li *et al*. 2008; Buffalari & See 2010). Human fMRI studies similarly report amygdala activation in response to heroin cues (Langleben *et al*. 2008; Mei, Zhang, & Xiao 2010; Li *et al*. 2012; Langleben *et al*. 2014), and pharmacological agents used to manage OD attenuate both amygdala activation and craving (Langleben *et al*. 2008; Mei *et al*. 2010; Langleben *et al*. 2014). However, whilst one recent meta‐analysis of cue‐elicited craving paradigms did identify an association between amygdala activation and subjective craving (Chase *et al*. 2011), another meta‐analysis did not (Kuhn & Gallinat 2011).

One potential reason for this discrepancy may relate to differences in the methodology employed to examine brain‐craving relationships. Traditional fMRI designs correlate average magnitude of BOLD signal during cue presentation with mean craving scores. This approach may be problematic given the non‐quantitative nature of BOLD, and the variation of craving over time. The current time course study allowed us to investigate how the shape of the BOLD signal related to subjective craving over time. Unlike a number of previous studies, we did observe a significant correlation, suggesting that this approach may be more reliable, and/or sensitive, in detecting a biomarker of subjective craving.

The effects within the amygdala were unilateral, possibly suggesting a left‐sided laterality for craving. Previous meta‐analyses of cue‐elicited craving studies have demonstrated a different lateralisation dependent upon the substance of dependence (Kuhn & Gallinat 2011) or the contrast investigated (Chase *et al*. 2011). Study design may also influence lateralisation, as the left amygdala is more commonly recruited in block designs rather than event‐related designs (Sergerie, Chochol, & Armony 2008), which may reflect different temporal dynamics (i.e. the right amygdala rapidly and automatically detects emotional stimuli, whilst the left amygdala engages in a more sustained evaluation) (Wright *et al*. 2001; Sergerie *et al*. 2008). Our sustained video stimulus would thus plausibly lead to a left‐lateralised response.

### Dorsal ACC and craving

The dorsal ACC region is involved in both affective processing and higher order cognitive and behavioural control (Yucel & Lubman 2007; Etkin, Egner, & Kalisch 2011; Sheth *et al*. 2012; Shenhav *et al*. 2013). Cue‐elicited ACC response has previously been observed in cocaine‐dependent individuals viewing a short (<5 minutes) cocaine video (Wexler *et al*. 2001), and positive correlations between ACC activity and craving have been reported (Kuhn & Gallinat 2011; Li *et al*. 2012). However, traditional fMRI designs typically do not allow separation of the generation of craving from attempts to control craving (Goldstein & Volkow 2011). Here, our time course analysis showed that the dACC was not significantly recruited during the video when craving was at its peak, but was engaged after the end of the video whilst craving was declining. This suggests that the dACC is not involved in the generation of craving, but may be recruited as newly abstinent individuals consciously attempt to bring craving under control. Indeed, nicotine craving studies have demonstrated that the dACC is recruited when participants ‘resist’ (Brody *et al*. 2007), or cognitively reappraise (Zhao *et al*. 2012), craving. Of particular interest here, it has been demonstrated previously that opioid users show inefficient dACC activation during inhibitory control (Yucel *et al*. 2007), which may reduce their ability to resist craving, and increase risk of relapse following detoxification. Future research should explore the relationship between dACC response in a newly abstinent group and duration of abstinence.

### Ventral medial prefrontal cortex, the ventral striatum and craving

Against expectations, we did not observe significant group or time effects for BOLD signal within the VmPFC and ventral striatum. The VmPFC is involved in the valuation of conditioned stimuli, signalling the subjective value of associated rewards (Elliott *et al*. 2008; Schoenbaum *et al*. 2009). VmPFC activation to drug cues has previously been observed, but typically in non‐treatment‐seeking individuals (Chase *et al*. 2011), suggesting that the lack of significant VmPFC recruitment in the current study may reflect altered motivational salience of drug cues in this newly abstinent group. It may also be possible that the vulnerability of the VmPFC to susceptibility artefact precluded the detection of significant VmPFC findings.

Extensive evidence implicates the ventral striatum in various aspects of reward processing (Haber & Knutson 2010) including reward prediction (O'Doherty *et al*. 2004) and goal‐directed action based on salient affective information (Corbit, Muir, & Balleine 2001; Corbit & Balleine 2011). It is therefore considered an important substrate of substance dependence, and we hypothesized that we would observe ventral striatum responses to the drug cue video; however, no such response was observed. As we argued for the VmPFC, this may relate to reduced motivational salience of cues in actively treated individuals in combination with increased engagement of cognitive control processes that have been shown to inhibit both VmPFC and ventral striatal response to drug cues (Volkow *et al*. 2010). However, meta‐analyses of previous fMRI studies report ventral striatum responses to drug cues in both treatment and non‐treatment‐seeking cohorts (Chase *et al*. 2011). Ventral striatal response to craving cues has also been observed in another study of inpatient detoxified opioid‐dependent individuals (Li *et al*. 2012). However, it is important to consider the reasons why people are in treatment. Drug detoxification is often compulsory in some countries (Werb *et al*. 2016) or may be ‘coerced’ (e.g. treatment to reduce or avoid a prison sentence); therefore, being ‘in treatment’ does not necessarily equate to ‘treatment seeking’. Different responses to drug cues may be expected in those who have voluntarily chosen treatment compared with those who have been compelled or coerced to enter treatment programmes. Furthermore, the exact stage of treatment may influence brain response to drug cues. In the current study, although 39 participants enrolled, approximately 50 percent self‐discharged from the detoxification unit before the fourth week of the residential stay when scanning took place. Therefore, it remains possible that our group, tested during the last week of a voluntary, recovery focussed inpatient treatment programme, represents a distinct ‘treatment‐seeking’ population maximally motivated to maintain abstinence. Longitudinal studies are needed to test hypotheses about differential responses to drug cues at different stages of dependence, treatment and abstinence (Jasinska *et al*. 2014).

### Limitations

There are a number of limitations of this study. Most of the OD group were still receiving psychoactive medications (Supporting Information Table S1). The limited numbers of participants did not allow exploration of the influence of medication; however, given its high prevalence within this population (Torrens, Gilchrist, & Domingo‐Salvany 2011; Vogel *et al*. 2013), inclusion of these individuals allows for a more representative study sample. Half of the OD participants were still receiving the opioid withdrawal medication lofexidine, and one small study reported that high doses of lofexidine (2.4 mg twice daily) reduce craving (Sinha *et al*. 2007). However, lofexidine is not generally considered to suppress psychological cravings (Gish *et al*. 2010), and a much lower dose was administered to participants at the point of their participation in the present study (0.2 mg twice daily). Furthermore, differences in IQ and years of education between our groups may have influenced findings. However, a particular strength to the study was our selection of healthy volunteers based upon occupational experience of drug use. We chose to prioritize this over matching for educational performance, which findings from our group suggest do not impact upon incentive motivation and reward processes (Bland *et al*. 2016).

Another limitation was that study numbers were relatively small, largely because this is a difficult group to recruit, especially given recent changes in UK addiction service provision (the inpatient unit admission criteria changed during the period of recruitment such that only those with complex health needs were admitted, resulting in many patients being ineligible for the study). This may have had an impact on results; e.g. the time course in the VmPFC did not reach significance because of high variability in this region prone to signal dropout. In a larger sample, this finding might have reached significance.

## Conclusion and Clinical Implications

Using a novel paradigm investigating dynamic brain and craving responses, we have demonstrated a similar time course that links the left amygdala with the emergence of subjective craving, suggesting a craving generative role for the amygdala. Amygdala response to drug cues may represent a persistent vulnerability to relapse within this cohort. By analysing brain and craving responses after the end of the craving video, we found increased dACC BOLD response only during the period of decreasing subjective craving. This suggests a role for the dACC in behavioural/cognitive control in recovering individuals following a period of cue‐induced craving. Persistent amygdala activation and craving in response to drug cues, together with inefficient dACC‐mediated control, may contribute to relapse following treatment. Promising cognitive and pharmacological treatments have been shown to reduce amygdala cue reactivity and increase dACC activity in both preclinical models and human tests of cognitive performance (Spence *et al*. 2005; Xue *et al*. 2012). Our time course‐based approach may represent an important biomarker for evaluating these treatments in dependent individuals.

## Conflict of Interest

The authors declare no conflicts of interest

## Authors Contribution

RE was responsible for study concept. RE, DIL, SM, IA, BD, PSB, LAP, AM and SH made an active contribution to the study design and concept. AM and QF recruited participants. AM collected the data. AM, SM and SH analysed the data. AM drafted the manuscript. RE, DIL, IA and BD provided critical revision of the manuscript for important intellectual content. All authors critically reviewed the content and approved the final version for publication.

## Supporting information


**Table S1.** **Psychoactive medication taken by opioid dependent participants during each session.** Number and proportion (in brackets) shown.
**Table S2. Results from the mixed ANOVAs on the drug cue‐neutral BOLD signal time course for the whole session (T**_**0**_ **‐T**_**15**_ **)** * = significant at *p*<0.05, ** = significant at *p*<0.05 Bonferroni corrected for the 6 ROIs.
**Table S3. Results from the mixed effect ANOVAs on the drug cue‐neutral BOLD signal for the video period only (T**_**1**_ **to T**_**10**_ **).** * = significant at *p*<0.05, ** significant at *p*<0.05 Bonferroni corrected for the 6 ROIs.
**Table S4. Results from the mixed effect ANOVAs on the drug cue‐neutral BOLD signal for the post‐ video period only (T**_**11**_ **to T**_**15**_ **)**. * = significant at *p*<0.05, ** significant at *p*<0.05 Bonferroni corrected for the 6 ROIs.
