# Supplementary material for: Time‐dependent neuronal changes associated with craving in opioid dependence: an fMRI study
Source: Addict Biol. 2017 Sep 22;23(5):1168–78. doi: 10.1111/adb.12554 (PMC6120475; doi:10.1111/adb.12554)
Supplement: Supplementary file 1 — Table S1. Psychoactive medication taken by opioid dependent participants during each session. Number and proportion (in brackets) shown. Table S2. Results from the mixed ANOVAs on the drug cue‐neutral BOLD signal time course for the whole session (T 0 ‐T 15 ) * = significant at p<0.05, ** = significant at p<0.05 Bonferroni corrected for the 6 ROIs. Table S3. Results from the mixed effect ANOVAs on the drug cue‐neutral BOLD signal for the video period only (T 1 to T 10 ). * = significant at p<0.05, ** significant at p<0.05 Bonferroni corrected for the 6 ROIs. Table S4. Results from the mixed effect ANOVAs on the drug cue‐neutral BOLD signal for the post‐ video period only (T 11 to T 15 ). * = significant at p<0.05, ** significant at p<0.05 Bonferroni corrected for the 6 ROIs. [file ADB-23-1168-s001.docx]

**Supplementary Materials**

| **Medication** | **Drug Cue Session** | **Neutral Session** |
| --- | --- | --- |
| Lofexidine | 8 (44%) | 9 (50%) |
| Diazepam | 2 (11%) | 2 (11%) |
| Nitrazepam | 1 (6%) | 1 (6%) |
| Promazine | 1 (6%) | 3 (17%) |
| Risperidone | 1 (6%) | 1 (6%) |
| Naltrexone | - | 1 (6%) |
| Fluoxetine | 1 (6%) | 1 (6%) |
| Sertraline | 1 (6%) | 1 (6%) |
| No medication | 5 (28%) | 7 (38%) |

**Table S1.** **Psychoactive medication taken by opioid dependent participants during each session.** Number and proportion (in brackets) shown.

|  | ***Time***  ***T_0_*  *to T_15_*_(crave-neut)_*)*** | | **Time*Group**  ***T_0_* *to T_15_*_(crave-neut)_*)*** | |  | |  | **Group**  ***T_0_* *to T_15_*_(crave-neut)_*)*** | | |
| --- | --- | --- | --- | --- | --- | --- | --- | --- | --- | --- |
|  | F-Value | *P*-value | F-Value | *P*-value | | df |  | F-Value | *P*-value | df |
| Anterior cingulate cortex | 3.78 | 0.004** | 1.63 | 0.164 | | 4.39,153.60 |  | 3.82 | 0.056 | 1, 35 |
| Ventral medial prefrontal cortex | 1.16 | 0.333 | 0.87 | 0.490 | | 4.44, 155.47 |  | 1.81 | 0.187 | 1, 35 |
| Left amygdala | 1.35 | 0.258 | 2.44 | 0.056 | | 3.60, 125.94 |  | 6.52 | 0.014* | 1, 35 |
| Right amygdala | 0.73 | 0.546 | 0.33 | 0.817 | | 3.18, 111.45 |  | 0.27 | 0.605 | 1, 35 |
| Left ventral striatum | 0.60 | 0.605 | 1.15 | 0.333 | | 2.81, 98.18 |  | 0.07 | 0.789 | 1, 35 |
| Right ventral striatum | 2.31 | 0.073 | 1.37 | 0.604 | | 3.37, 117.93 |  | 0.04 | 0.843 | 1, 35 |

**Results Tables**

**Table S2. Results from the mixed ANOVAs on the craving-neutral BOLD signal time course for the whole session (T_0_-T_15_)** * = significant at *p*<0.05, ** = significant at *p*<0.05 Bonferroni corrected for the 6 ROIs.

|  | ***Time***  ***T_1_*  *to T_10_*_(crave-neut)_*)*** | | **Time *Group**  ***T_1_* *to T_10_*_(crave-neut)_*)*** | |  | |  | | **Group**  ***T_1-_T_10_*_(crave-neut)_*)*** | | |
| --- | --- | --- | --- | --- | --- | --- | --- | --- | --- | --- | --- |
|  | F-Value | *P*-value | F-Value | *P*-value | | df | |  | F-Value | *P*-value | df |
| Anterior cingulate cortex | 1.36 | 0.251 | 1.213 | 0.308 | | 4.11, 143.9 | |  | 3.062 | 0.089 | 1, 35 |
| Ventral medial prefrontal cortex | 1.81 | 0.132 | 0.947 | 0.437 | | 3.88, 135.84 | |  | 2.683 | 0.110 | 1,35 |
| Left amygdala | 1.32 | 0.272 | 3.43 | 0.017* | | 3.26, 114.23 | |  | 7.960 | 0.007** | 1, 35 |
| Right amygdala | 1.23 | 0.300 | 0.456 | 0.762 | | 3.89, 135.98 | |  | 00.27 | 0.607 | 1, 35 |
| Left ventral striatum | 0.39 | 0.774 | 1.10 | 0.355 | | 3.17, 111.05 | |  | 0.597 | 0.445 | 1, 35 |
| Right ventral striatum | 2.560 | 0.051 | 0.335 | 0.824 | | 3.4, 119.20 | |  | 0.009 | 0.927 | 1, 35 |

**Table S3. Results from the mixed effect ANOVAs on the craving-neutral BOLD signal for the video period only (T_1_ to T_10_).** * = significant at *p*<0.05, ** significant at *p*<0.05 Bonferroni corrected for the 6 ROIs.

|  | ***Time***  ***T_11_*  *to T_15_*_(crave-neut)_*)*** | | **Time *Group**  ***T*_11_ *to T_15_*_(crave-neut)_*)*** | |  |  | **Group**  ***T_11_ to T_15_*_(crave-neut)_*)*** | | |
| --- | --- | --- | --- | --- | --- | --- | --- | --- | --- |
|  | F-Value | *P*-value | F-Value | *P*-value | df |  | F-Value | *P*-value | df |
| Anterior cingulate cortex | 1.03 | 0.385 | 1.38 | 0.252 | 3.23, 113.1 |  | 4.27 | 0.046* | 1, 35 |
| Ventral medial prefrontal cortex | 1.15 | 0.822 | 1.031 | 0.385 | 3.22, 98.03 |  | 0.43 | 0.519 | 1, 35 |
| Left amygdala | 2.91 | 0.041* | 3.60 | 0.018* | 2.83, 98.96 |  | 2.23 | 0.144 | 1, 35 |
| Right amygdala | 0.30 | 0.819 | 0.70 | 0.551 | 2.90, 101.67 |  | 0.21 | 0.643 | 1, 35 |
| Left ventral striatum | 2.45 | 0.073 | 0.564 | 0.626 | 2.77, 96.84 |  | 0.12 | 0.730 | 1, 35 |
| Right ventral striatum | 2.01 | 0.124 | 2.009 | 0.448 | 2.693, 94.27 |  | 0.13 | 0.720 | 1, 35 |
|  |  |  |  |  |  |  |  |  |  |

**Table S4. Results from the mixed effect ANOVAs on the craving-neutral BOLD signal for the post- video period only (T_11_to T_15_)**. * = significant at *p*<0.05, ** significant at *p*<0.05 Bonferroni corrected for the 6 ROIs.
